# Lipocalin 2 in cerebrospinal fluid as a marker of acute bacterial meningitis

**DOI:** 10.1186/1471-2334-14-276

**Published:** 2014-05-20

**Authors:** Tamazoust Guiddir, Ala-Eddine Deghmane, Dario Giorgini, Muhamed-Kheir Taha

**Affiliations:** 1 Institut Pasteur, Invasive Bacterial Infections Unit and National Reference Centre for Meningococci, 28 Rue du Dr Roux, 75724 Paris, Cedex 15, France

**Keywords:** Meningitis, Lipocalin 2, Inflammation, Cerebrospinal fluid, Diagnosis

## Abstract

**Background:**

Early differential diagnosis between acute bacterial and viral meningitis is problematic. We aimed to investigate whether the detection of lipocalin 2, a protein of the acute innate immunity response, may be used as a marker for acute bacterial meningitis.

**Methods:**

Transgenic mice expressing the human transferrin were infected by intraperitoneal route and were imaged. Cerebrospinal fluid (CSF) was sampled up to 48hours post- infection to measure lipocalin 2. We also tested a collection of 90 and 44 human CSF with confirmed acute bacterial or acute viral meningitis respectively.

**Results:**

Lipocalin 2 was detected after 5 h in CSF during experimental infection in mice. Lipocalin 2 levels were significantly higher (*p* < 0.0001) in patients with confirmed acute bacterial meningitis (mean 125 pg/mL, range 106–145 pg/mL) than in patients with acute viral meningitis (mean 2 pg/mL, range 0–6 pg/mL) with a sensitivity of 81%, a specificity of 93%, a positive predictive value of 96% and a negative predictive value of 71% in diagnosing acute bacterial meningitis.

**Conclusions:**

Increased levels of lipocalin 2 in cerebrospinal fluid may discriminate between acute bacterial and viral meningitis in patients with clinical syndrome of meningitis.

## Background

Acute bacterial meningitis (ABM) is a major cause of morbidity and mortality worldwide. The Wold Health Organization (WHO) estimates that there are around 1 million of cases per year worldwide with 135–200,000 fatal cases [1]. *Haemophilus influenzae* type b (Hib), *Streptococcus pneumoniae* (Sp), and *Neisseria meningitidis* (Nm) are the most frequent agents of ABM. Other agents are also incriminated in infants such as *Streptococcus agalactiae* and *Escherichia coli* K1. ABM is a medical emergency and requires immediate management that relies mainly on appropriate and prompt antibiotic treatment. However, the major differential diagnosis of ABM is acute viral meningitis (AVM) that does not require antibiotics and are usually of better prognosis. Laboratory confirmation requires lumber puncture and analysis of the cerebrospinal fluid (CSF). Etiologic diagnosis of ABM is performed by culture and non-culture methods (smear detection, nucleic acid detection by PCR and/or antigen detection by agglutination kits) [2]. Recognition of ABM is therefore needed at the admission and accurate diagnosis is mainly based on examination of CSF. Pleocytosis with predominance of polymorphonuclear leukocytes (PMN), low level of glucose in CSF with low CSF-blood glucose ratio and increase in CSF protein levels are usually encountered in ABM. However, overlapping values between ABM and AVM are reported.

While 71% of patients with ABM may have a leukocyte count of ≥1000 cells/μl in CSF samples, 15% of patients with AVM may display similar counts in CSF and up to 10% of ABM cases may show leukocyte counts of <100 cells/μl [3,4]. Cut-off values differed among studies with variable performances [5]. Analysis of CSF in meningitis may allow discriminating ABM from AVM and guide the decision to administer (or not) antibiotics [6,7]. Scores were developed for this purpose combining both clinical signs and biological markers such as serum levels of C-reactive protein (CRP) and procalcitonin (PCT) [4,8,9].

The lipocalin 2 (LCN2) is a small protein of 22 kDa involved in iron homeostasis that allows an alternative method to transferrin to deliver iron to the cytoplasm [10]. LCN2 was initially discovered in the granules of the polymorphonuclear cells and was called *Neutrophil Gelatinase Associated Lipocalin* (NGAL) [11]. LCN2 is a part of the acute innate immune response to bacterial infection. It allows sequestrating iron through interfering with siderophore-mediated iron acquisition by bacteria [12,13]. We have recently reported transcriptomic analysis in an experimental sepsis in transgenic mice expressing the human transferrin [14]. Several differentially expressed transcripts (DETs) corresponding to acute phase proteins were detected. Interestingly, one of these proteins, LCN2, was overexpressed in the brain of infected mice after 6 h of infection. LCN2 was also reported as an acute phase protein to be produced at the blood brain barrier by the choroid plexus epithelial cells and the endothelial cells of blood vessels [15]. We aimed to explore the detection of LCN2 in CSF as a marker of acute bacterial meningitis.

## Methods

### Clinical samples and patients

One hundred thirty four cerebrospinal fluids (CSF) addressed to the National Reference Center for Meningococci (NRCM) for molecular diagnosis of bacterial meningitis were tested. Available epidemiological data (age, sex), clinical and biological data, C-reactive protein (CRP) in blood, CSF levels of protein and glucose were analyzed. Confirmed ABM was defined as more than 100 leukocytes in CSF and the detection of bacteria (culture, PCR, slide agglutination or positive smear detection). PCR-based diagnosis was performed as previously described [16]. Enterovirus PCR was performed in hospitals that sent CSF samples to the NRCM.

### Generation of the bioluminescent LNP24198lux strain

*N. meningitidis* strain LNP24198 is a clinical isolate of serogroup C, serotype 2a and serosubtype P1.7,1 (PorA VR1 = 7-1 and VR2 = 1) that belongs to the clonal complex ST-11 [17]. The plasmid pXen-13 containing *Photorhabdus luminescence luxCDABE* operon was purchased from Xenogen Corp., Alameda, CA and was modified by insertion of a *Neisseria* specific promoter sequence. A 600 bp promoter sequence of the meningococcal *porB* gene (PporB) was PCR amplified using primers PorB3 (5′-GGTGCTGAAGCACCAAGTGA -3′) and PorB4 (5′- GGCAATCAGGGATTTTTTCA-3′) and subcloned into a *Bam*HI site upstream of the *luxCDABE* operon to express the *luxCDABE* operon under the control of the PporB meningococcal promoter *N. meningitidis*. The generated plasmid was named pDG33. The fragment encompassing the *luxCDABE* cassette and the *porB* promoter was extracted by digesting pDG33 with KpnI and SacI restriction enzymes and inserted into *Bam*HI site of the plasmid pTE-KM [18], upstream the kanamycin *aph3’* resistance cassette. The resulting vector was named pDG34 in which, the PporB-*luxCDABE*-*aph3’* was flanked by the meningococcal *pilE* gene and 120 bp downstream *pilE* gene to facilitate the recombination upon transformation to obtain the LNP24198lux strain.

### Animal infection, analysis and imaging studies

We have previously described the use of transgenic mice expressing the human transferrin infected by intraperitoneal route (ip) as an experimental model of meningococcal infection [19]. Mice were in-house bred and were kept in a biosafety containment facility, in filter-topped cages with sterile litter, water and food, according to institutional guidelines.

Each mouse was infected with standardized inocula of the bioluminescent strain LNP 24198lux. Bacterial infection images were acquired using an IVIS® 100 system (Xenogen Corp., Alameda, CA) according to instructions from the manufacturer and as previously described [14]. Analysis and acquisition were performed using Living Image® 3.2 software (Xenogen Corp.). Images were acquired using 1 min of integration time with a binning of 16. All other parameters were held constant. Quantification was performed using the total photons per second emitted by each mouse after 30 min, 2 h, 5 h and 24 h of infection by defining regions of interest. An uninfected mouse under the same conditions of acquisition was used to define the background. After each imaging point, CSF samples (10 μl) were collected in a 30 μl 0.9% NaCl from each mouse by puncture from the cisterna magna as previously described [20]. Serial dilutions of CSF samples were plated on GCB medium supplemented with Kellogg supplements to determine the number of colony forming units (CFU).

### Ethics statement

This study was carried out in strict accordance with the European Union Directive 2010/63/EU (and its revision 86/609/EEC) on the protection of animals used for scientific purposes. Our laboratory has the administrative authorization for animal experimentation (Permit Number 75–1554) and the protocol was approved by the Institut Pasteur Review Board that is part of in the Regional Committee of Ethics of Animal Experiments of Paris region (Permit Number: 99–174). CSF samples were initially received for diagnosis that is part of the primary management of suspected meningococcal meningitis. The patients were informed on the secondary use of CSF samples for research and they gave their consent for this use. The printed form of this informed consent is sent to our laboratory. This procedure is performed according to the French public health code (Art L1211-2).

### Western blot and Enzyme-linked immunosorbent assay (ELISA)

Thirty micro liters of human CSF, 15 μl of diluted CSF (see above) or 2 μl of blood from infected mice were separated in 14% SDS- polyacrylamide gels and then transferred onto nitrocellulose membrane. Western blotting was performed as previously described [21] using anti-human or anti-mouse lipocalin 2 antibody (Abcam, Cammbridge, UK). Densitometry was performed using Image J® software (http://imagej.nih.gov/ij/). All density data were corrected for the background by subtraction of the density of the negative control (confirmed non bacterial meningitis). Results were then expressed as a ratio of the corrected density obtained for each CSF over the corrected density obtained for the positive control (confirmed acute bacterial meningitis). ELISA was performed with *lipocalin2/NGAL Human ELISA kit®* (Abcam, Cambridge, UK) according to the manufacturer’s recommendations. The expression of LCN2 gene in mice was performed by reverse-transcriptase-PCR (RT-PCR) analysis as previously described [22].

### Statistical analysis

Qualitative data were analyzed using the Chi-square test. Statistically significant differences were assumed when p < 0.05. Geometric means as well as lower and upper 95% confidence intervals were calculated using GraphPad InStat® version 3.06 (GraphPad Software, San Diego, CA, USA). Specificity, sensitivity, positive predictive value, negative predictive value and likelihood ratios were calculated as previously described [23].

## Results

### Analysis of lipocalin 2 expression during experimental meningococcal infection

**Figure 1:**
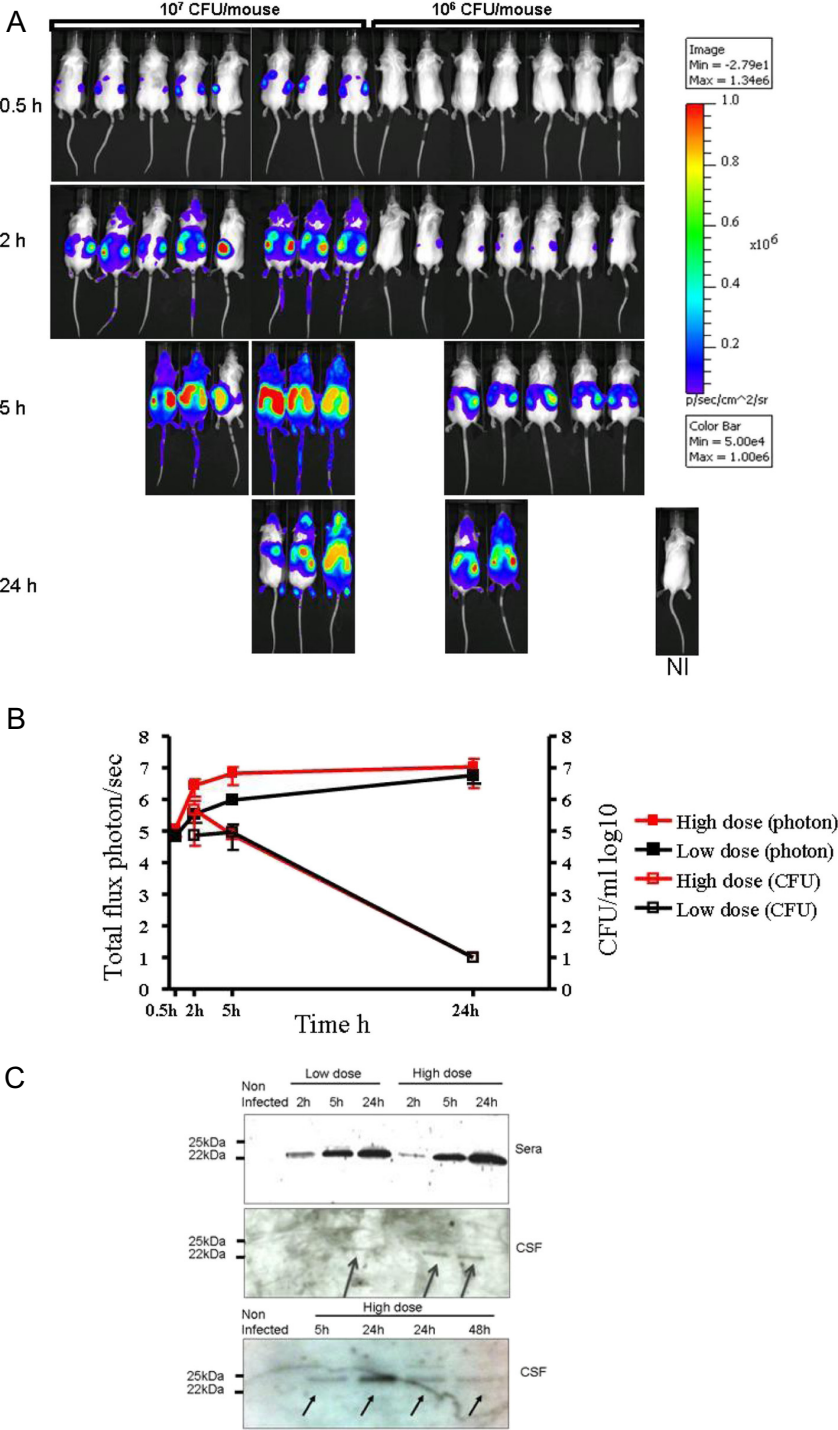
**Dissemination of *N. meningitidis *in BALB/c mice that were infected by intraperitoneal injection of 10**^**6**^ **CFU (low dose) or 10**^**7**^ **CFU per mouse (high dose) of *N. meningitidis *strain LNP 24198lux expressing the luciferase.** Mice were analyzed for bioluminescence at the indicated times. Images depict photographs overlaid with colour representations of luminescence intensity, measured in photons/s-cm2-sr and indicated on the scales, where red is most intense and blue is least intense. (Top row) **(A)** Dorsal views of 8 mice (high dose) and 7 mice (low dose). A non infected mouse (NI) is also shown **(B)**. The luminescence was quantified and expressed as means ± SD from each category at the indicated times by defining specific representative region of interest encompassing the entire animal. **(C)** Western blotting of CSF and blood from mice to detect lipocalin 2 (arrows) after the indicated time points of infection and using 10^6^ CFU (low dose) or 10^7^ CFU per mouse (high dose) as in **(A)**.

We first explored whether lipocalin 2 was detectable in CSF from infected mice during experimental infection as expected from the previously reported overexpression of lipocalin 2 specific transcripts in brain [14]. Two groups of 7 and 8 BALB/c female mice were infected intraperitoneally by 10^6^ CFU/mice or 10^7^ CFU/mice of the strain LNP24198lux respectively and 3 uninfected mice were used as controls. Mice were imaged at 30 min, 2 h, 5 h and 24 h (Figure 1A). Two or three mice from each bacterial dose were anesthetized at 2 h, 5 h and 24 h of infection and CSF samples were withdrawn (see Material and Methods). Bacterial counts in CSF were determined by serial plating (Figure 1B) and lipocalin 2 was detected in CSF samples by Western blotting (Figure 1C). Bioluminescent signals remained localized at the injection site 30 minutes after the bacterial infection but spread rapidly after 2 hours particularly in mice that received the highest bacterial dose. The total emitted photons on the skulls increased and paralleled the increase in the bacterial counts in CSF and in blood (Figure 1B and data not shown). In CSF, bacteria were detected at 2 h of infection and lipocalin 2 was detected at 5 h of infection in mice that received the highest bacterial dose and at 24 h for both doses of infection. At these points, very few leukocytes were detected in CSF of infected mice (data not shown). The detection of lipocalin 2 in blood also matched that in CSF (Figure 1).

The experiment was repeated using the 10^7^ CFU/mice dose and CSF samples were taken up to 72 hours of infection. Lipocalin 2 was again detectable at 5 h after infection, reached a peak at 24 h then decrease and was no more detectable at 72 h (Figure 1C and data not shown).

RT-PCR performed on RNA extracted from the brain of infected and non-infected mice confirmed the induction of the gene encoding the lipocalin 2 as previously described [14]. This induction was also observed in blood (data not shown). All these results confirm the induction and the early detection of lipocalin 2 in the CSF after bacterial infection in mice.

### Characterization and classification of human CSF from clinical samples

**Figure 2:**
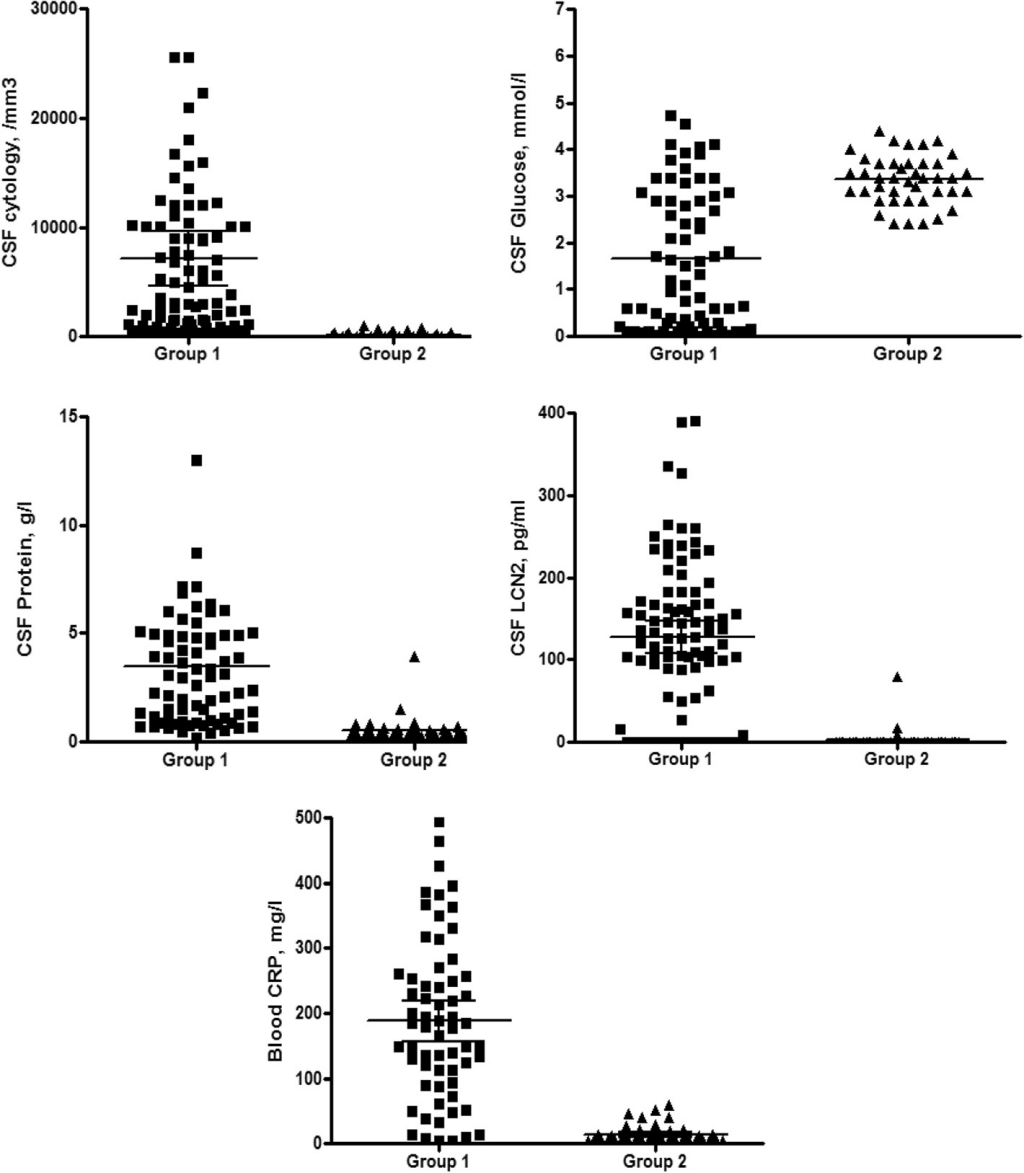
**Cerebrospinal fluid levels of LCN2, cytology, glucose, protein and blood CRP in the two groups.** Median values and 95% confidence intervals are given.

We next aimed to test whether lipocalin 2 can be detected in CSF from patients with acute bacterial meningitis. We explored a large collection of CSF samples (n = 134) that are received at the National Reference Center for Meningococci for non-culture diagnosis by PCR. The samples were classified in two groups: The first group (group 1) included 90 CSF (67%) that showed more than 100 leukocytes and were positive for at least one of the following tests: PCR, culture, smear detection or slide agglutination. *N. meningitidis* (Nm) was the main bacterial agent that was detected in our samples (n = 88) due to the fact that samples were tested for suspected meningococcal meningitis. One sample was positive for *S. pneumoniae* (Sp) and another was positive for *Streptococcus* group A. This group corresponded to confirmed bacterial meningitis. Nm positive samples were of several groups; 73% NmB (n = 66), 12% NmC (n = 11), 7% NmW (n = 6), 6% NmY (n = 5). The multi locus sequence typing (MLST), that determines the clonal complex, was performed for 45 (51% of Nm positive samples): 10 belonged to the hyperinvasive clonal complex ST-11 and 35 belonged to other clonal complexes. The second group (group 2) included 44 CSF that corresponded to PCR-confirmed acute enteroviral meningitis. The characteristics of patients and CSF of each of these two groups are presented in Table 1. In spite of overlapping values, biological markers of acute bacterial meningitis differed significantly between group 1 and group 2. In particular, known biological markers for ABM were present such as high numbers of leucocytes (mean 7133), and high proportion of neutrophils (mean 87%), high protein levels (mean 3.4 g/L) and low levels of glucose in CSF (mean 1.6 mmol/L) and CRP in blood (mean 188 mg/L) (Table 1 and Figure 2).

**Table 1:** Characteristics of patients and CSF tested

	**Group 1**		**Group 2**		
	**Confirmed acute bacterial meningitis n = 90**		**Confirmed acute viral meningitis n = 44**		
	**Mean**	**IC 95%**	**Mean**	**IC 95%**	**p**
**Age, y**	21	17-25	4.5	3-6	<0.0001
**CSF cytology, mm**^**3**^	7133	4625-9642	160	90-230	<0.0001
**CSF PMN, %**	87	83-91	46	35-57	<0.0001
**CSF Protein, g/L**	3.4	2.7-4.2	0.5	0.3-0.7	<0.0001
**CSF glucose, mmol/L**	1.6	1.3-2	3.3	3.2-3.5	<0.0001
**Blood CRP, mg/L**	188	157-219	14	9-18	<0.0001
**CSF LCN2, pg/mL**	127	108-146	2.4	0-6.2	<0.0001

### Analysis of levels of lipocalin 2 in human CSF

**Figure 3:**
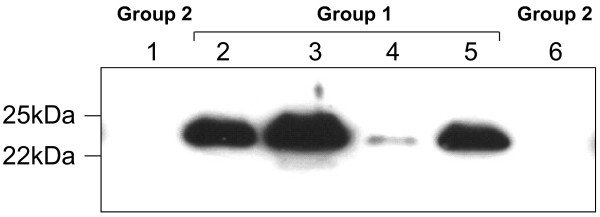
**Western blotting on human CSF with anti-LCN2 antibody.** Data are from individual CSF samples from groups 1 and 2. Molecular markers are indicated on the left. Results of meningococcal detection and the corresponding group of tested CSF are indicated. Group 1 (confirmed acute bacterial meningitis) and Group 2 (confirmed acute viral meningitis).

We next tested all the 134 CSF samples from the two groups by Western blotting using antibodies against human LCN2 and using an ELISA kit (Abcam, Cambridge, UK). Western blotting showed the expected band with an apparent molecular mass of 22 kDa that was observed with variable intensities (Figure 3). Western blots and ELISA were analysed as described in Material and Methods section after normalization with the negative and positive controls. Data from ELISA and Western blotting showed significant variations (p < 0.0001) in the levels of LCN2 that were detected in CSF among the two groups of CSF. CSF from group 1 (acute bacterial meningitis) showed higher levels of LCN2 than group 2 (acute viral meningitis) (Table 1). We also compared the two groups for other biological markers of inflammation: the blood level of CRP and CSF markers of acute bacterial meningitis such as high levels of protein and low levels of glucose. These comparisons also corroborated the levels of LCN2 in CSF in group 1 (confirmed ABM) compared to group 2 (confirmed AVM) (Table 1 and Figure 2). As a biological marker of acute bacterial meningitis, the detection of lipocalin 2 in CSF has a sensitivity of 81%, a specificity of 93%, a positive predictive value of 96% and a negative predictive value of 71%. The likelihood ratio of a positive test result is 11.6 (the sensitivity divided by 1- specificity). The likelihood ratio of a negative test result is 0.2 (1- sensitivity divided by specificity).

All these data taken together, strongly suggest that lipocalin 2 can be used as a marker to help confirming acute bacterial meningitis.

## Discussion

Reliable tests that allow early discrimination between ABM and AVM are still lacking, and biological findings in blood and CSF are quite similar and indecisive at the onset of the disease. Indeed, 12 hours of illness may be required before CRP to increase above normal levels in ABM [24]. Moreover, CRP may also increase in acute viral meningitis [4,25]. PCT also requires 6 hours to increase in ABM [26]. Pleocytosis and neutrophil predominance usually characterize ABM but they can be observed in AVM and may be absent in about 10% of ABM [27]. In a retrospective study, leukocyte counts in viral meningitis ranged between 42 and 320 per mm^3^ with a mean of 110 per mm^3^[28] compared to the mean of 160 per mm^3^ in our study. These markers associated with clinical signs and symptoms may be sensitive in recognizing ABM but may still lack specificity and hence do not allow avoiding unnecessary antibiotic treatment [29]. Other markers were also tested to discriminate AVM from ABM such as the soluble triggering receptor expressed on myeloid cells-1 (sTREM-1), haemoglobin scavenger receptor (CD163) and High Mobility Group Box 1 (HMGB1). As for CRP and PCT, these markers are produced during the acute phase of inflammation in response to release a large number of released bacterial components such as lipopolysaccharide (LPS) and peptidoglycan (PG) [30]. These markers may be of interest to evaluate the severity of serious bacterial systemic infections [31,32]. The concentrations of inflammatory cytokines such as TNF-alpha, IL-1beta and IL-8 in the CSF were also analysed in the differential diagnosis of meningitis [33,34] but cannot be used in routine practice. Recently, heparin-binding protein (HBP) was also suggested as a marker for acute bacterial meningitis as HBP was shown to increase in CSF in patients with ABM but the earliness of this increase was not evaluated [35]. In spite of plethora of markers, the question of discriminating AVM from ABM is still debated and how to make the decision of rapid administration of broad-spectrum antibiotics is still open.

LCN2 has a bacteriostatic effect as it is able to sequester siderophores that are essential for bacterial survival [12,13,36]. Lipocalin 2 knock-out mice succumbed rapidly after intraperitoneal infection of *Escherichia coli*, in contrast to wild-type mice [12]. Lipocalin 2 is an acute phase protein and an actor of the innate immunity [37]. It has multifaceted roles and is involved in several pathologies [38]. Our study in mice suggests that lipocalin 2 is not detectable in CSF of non infected mice and can be detected as early as 5 h after infection and before the pleocytosis. This may be due to the induction of the expression of LCN2 from epithelial cells of the choroid plexus [15]. Our imaging data in mice are compatible with this explanation as bioluminescent bacteria were clearly located in the skull and bacteria were detected in CSF. The highest levels of LCN2 were thereafter detected at 24 h when pleocytosis may also amplify the levels of LCN2 in CSF.

Variations in the level of LCN2 were observed among patients with confirmed ABM. Early antibiotic treatment (n = 38 in group 1 of our collection) and/or corticosteroids may modify LCN2 production. The timing of lumbar puncture may also of importance in interpreting CSF findings. Moreover, the long conservation of CSF samples in the tested collection as well as the conditions of this conservation may also be responsible for the low detection of LCN2 in true ABM cases. These considerations may explain the relative low NPV (71%) in our study. Under these conditions, the high likelihood ratio of a positive result (11.6) indicates that a positive test multiplies the pre-test odds by a factor of 11.6. This means that the test is better at ruling in a condition than ruling it out. This would be of interest in case the incidence of acute bacterial meningitis is to be reduced after the introduction of vaccines targeting agents of ABM. The detection of lipocalin 2 in sera from infected mice may allow discrimination when CSF was not collected or lumber puncture was not feasible.

One limitation of our study is that the samples were studied retrospectively and that the samples were enriched for ABM (mainly meningococcal ABM) as there were sent to the NRCM for suspicion of meningococcal meningitis. The development of a rapid test (for example a dipstick test) will open the possibility to perform a multisite study with prospective inclusion of patients suffering of clinical syndrome of meningitis. The performance of such a direct and rapid test for LCN2 detection in CSF and blood will be compared to that of other markers of ABM under the conditions of “in routine” management of ABM.

## Conclusion

Our data clearly indicate that LCN2 levels in CSF are highly increased in CSF in patients with ABM. We suggest that LCN2 detection in CSF from patients with clinical meningitis may help in differential diagnosis between acute bacterial and viral meningitis and may improve decision making for treatment algorithms in meningitis.

## Competing interests

The authors declare no conflict of interest.

## Authors’ contributions

TG, AED and MKT participated in the study design and the preparation of the manuscript, TG, AED, DG and MKT participated in the laboratory experimental work and in the interpretation of data. All authors read and approved the final manuscript.

## Pre-publication history

The pre-publication history for this paper can be accessed here:

http://www.biomedcentral.com/1471-2334/14/276/prepub
